# SCANDARE: an institutional dynamic prospective interventional biobanking study

**DOI:** 10.1186/s12885-026-15680-5

**Published:** 2026-02-05

**Authors:** Constance Lamy, Léonard Laurent, Olivier Choussy, Nicolas Pouget, Fabrice Lecuru, Fabien Reyal, Anne-Sophie Plissonnier, Amir Kadi, Frédérique Berger, Joey Martin, Antoine Dubray-Vautrin, Grégoire Marret, Edith Borcoman, Emanuela Romano, Marie-Paule Sablin, Luc Cabel, Camille Pasquesoone, Olivier Lantz, Delphine Louis, Doriane Gorret, Ines Dias Da Silva, Odette Mariani, Olivier Delattre, Ivan Bièche, Nicolas Servant, Manuel Rodrigues, Jean-Yves Pierga, Romain-David Seban, Elisabetta Marangoni, Antonin Morillon, Clotilde Théry, Charlotte Proudhon, Eliane Piaggio, Céline Vallot, Fatima Mechta-Grigoriou, Julie Flavius, Célia Dupain, Géraldine Gentric, Anne Vincent-Salomon, Maud Kamal, Christophe Le Tourneau

**Affiliations:** 1 Department of Drug Development and Innovation (D3i), Paris-Saclay University, Paris, France; 2 Department of Head and Neck Surgery, Institut Curie, Paris, France; 3 Department of Breast, Gynecological and Reconstructive Surgery, Institut Curie, Université de Paris Cité, Paris and Saint-Cloud, France; 4 DREH Pôle Promotion, Institut Curie, Saint-Cloud, France; 5 Department of Biometry, Institut Curie, Saint-Cloud, France; 6 Department of Pathology, Oscar Lambret Cancer Centre, Lille, France; 7 Clinical Immunology Laboratory, Institut Curie, Paris, France; 8 Centre d’investigation Clinique en Biothérapie Gustave-Roussy Institut Curie (CIC-BT1428), Paris, France; 9 Department of Pathology, Centre Des Ressources Biologiques, Institut Curie, Paris, France; 10 Department of Genetics, Institut Curie, Paris, France; 11 Computational Oncology, PSL Research University, Mines Paris Tech, INSERM U1331, Paris, 75005 France; 12 Bioinformatics Platform, Institut Curie, Mines Paris Tech, Paris, France; 13 Department of Medical Oncology, Institut Curie, Paris & Paris Cité, France; 14 INSERM U830, DNA Repair and Uveal Melanoma (D.R.U.M.), Equipe Labellisée Par la Ligue Nationale Contre Le Cancer, Institut Curie, PSL Research University, Paris, France; 15 Department of Nuclear Medicine, Institut Curie, Saint-Cloud, 92210 France; 16 Translational Research Department, Institute of Curie, Paris, France; 17 Institut Curie, CNRS UMR3244, Sorbonne University, PSL University, Paris, France; 18 Institut Curie, INSERM U932 and Curie CoreTech Extracellular Vesicles PSL Research University, Paris, France; 19 Circulating Tumor Biomarkers Laboratory, SiRIC, Translational Research Department, Institut Curie, PSL Research University, Paris, 75005 France; 20 Univ Rennes, Inserm, EHESP,, Irset (Institut de recherche en santé, environnement et travail) - UMR_S 1085, Rennes, France; 21 Institut Curie, PSL University, Department of Translational Research, Inserm U932, Laboratory of Immunity and Cancer, Paris, F-75005 France; 22 Stress and Cancer Laboratory, Institut Curie, INSERM, U830, PSL Research University, Ligue Nationale Contre le Cancer labeled Team, Paris, France; 23 Department of Pathology, Institut Curie, PSL University, INSERM U934, CNRS UMR 3215, Paris, France; 24 National PRecISion Medicine Center in Oncology, Gustave Roussy Cancer Campus, Paris, France

**Keywords:** Biobanking Study, Oncology, Precision Medicine

## Abstract

**Purpose:**

We set up a prospective longitudinal biobanking study answering scientific questions within a regulatory framework and patient-centered approach. We aim in this paper to present the opportunities of such a study, as well as the challenges and the limitations.

**Patients and methods:**

SCANDARE (NCT03017573) is an institutional first monocentric then multicentric biobanking study. The study enrolled adult patients with newly diagnosed head and neck squamous cell carcinoma, triple negative breast cancer, ovarian and cervical cancer. All patients signed a consent form before any procedure. Tumor tissue and blood samples are collected at several time points during patient's journey, including at diagnosis, post-neoadjuvant chemotherapy in case of neoadjuvant treatment, at surgery, at recurrence and at disease progression following treatment initiated at recurrence. Clinical data are entered into an eCRF, whereas generated data are centralized in a secure database.

**Results:**

SCANDARE started in 2017 at Institut Curie and has included 676 patients at date. SCANDARE successfully addressed challenges related to patient consent, regulatory compliance, and logistical integration of sample collection into routine clinical practice. The study facilitated the longitudinal collection and preservation of tumor and blood samples, enabling comprehensive analyses from histopathology to genomics and proteomics needed for the 35 ongoing projects run by academic research groups and industry. The implementation of optimized sampling and data workflows enabled high-quality data. Several other cohorts are planned to open soon.

**Conclusion:**

SCANDARE is an institutional, prospective and dynamic biobanking study that successfully enabled the implementation of 35 research projects with clinical and omics data centralized in a secure database available for all collaborators on demand. Looking ahead, SCANDARE aims to expand its scope by including additional cancer types and patient cohorts, further enhancing the potential for translational research and personalized medicine in oncology.

**Supplementary Information:**

The online version contains supplementary material available at 10.1186/s12885-026-15680-5.

## Introduction

The advent of precision oncology has gradually permeated our daily practice. Mainly due to the rapid evolution of Next Generation Sequencing techniques, precision oncology became a critical turning point for modern oncology therapeutic strategies. This is highlighted by the accelerated approval in the last decade of multiple targeted therapies and immunotherapies [1]. For example, drugs targeting ALK in *ALK*-translocated non-small cell lung cancer and drugs targeting PARP for advanced *BRCA* mutated breast cancer improved overall survival as compared to cytotoxic chemotherapy [2, 3]. Tissue-agnostic therapeutic strategies based on predictive biomarkers and signatures also emerged, such as immune checkpoint inhibitors for Tumor Mutational Burden (TMB) high or Microsatellite Instability (MSI) tumors [4–6].

Precision oncology relies today on translational research that allow effectively bridging fundamental research to the clinic [7]. The development of patient-derived models was reported to accelerate the development of novel targeted therapies (e.g. colorectal cancer) [8], and the discovery of predictive biomarkers [9, 10]. To fuel this translational research, the development of biobanking studies following the good clinical practice guidelines has been instrumental. Biobanking studies are designed to collect biological, clinical and imaging data in order to allow researchers to answer their scientific questions. The ideal biobanking study provides a longitudinal, extensive, comprehensive samples collection along with curated clinical data throughout patients’ journey to researchers. The complex interweaving of clinical and fundamental research must be facilitated by a standardized workflow and operating procedures.

Conducting biobanking studies comes with several challenges mainly centered on regulatory bottlenecks, data protection and patients’ enrollment in the absence of any therapeutic intervention. Despite these challenges and the initial investment required, translational medicine continues to grow. Initiatives like "All of Us" aims to collect data from one million people to improve health research while "Cancer Moonshot" seeks to reduce cancer mortality through extensive research and innovation [11, 12]. These efforts are mirrored in Europe by projects like, the lung cancer biobank at Heidelberg center [13], the dynamic biobank coordinated in London Barts cancer institute [14], TuBaFrost tumor biobanking coordinated by Organization of European Cancer Institutes (OECI) [15], the German (BiO)-project in ovarian and breast tumors [16] and the cervical cancer prospective biobanking study, Bio-RAIDs [17]. All emphasizing the global commitment to enhancing precision oncology and translational research, including studies on intratumoral heterogeneity, circulating tumor DNA (ctDNA) or tumoral microenvironment and immune systems [18–20].

We present here the SCANDARE biobanking study, an initiative of Institut Curie, Paris and Saint-Cloud, France that started in January 2017 (NCT03017573). SCANDARE aims to longitudinally collect biological samples and clinical data from diagnosis onwards for immunological and genomics analyses. We will describe how SCANDARE was set up and the challenges we faced before establishing a structure that now supports 35 research projects run in collaboration with academic and industry research teams.

Compared with existing initiatives, SCANDARE uniquely combines a broad research scope, longitudinal sampling from diagnosis to disease evolution, multiple biospecimen types and preservation strategies, and access to high-quality, structured clinical and molecular data to support diverse translational research projects.

## Patients and methods

### Study design and objectives

SCANDARE (NCT03017573, 11/01/2017) is a dynamic prospective and multicentric biobanking study initiated by Institut Curie, Paris and Saint-Cloud, France. The broad primary objective of SCANDARE is to elucidate the relationship between tumor molecular alterations, the microenvironment and immune parameters, based on biological and curated clinical data collected at key clinical timepoints during patients’ disease evolution. As knowledge constantly evolves, the study is tailored to answer scientific questions raised by researchers.

Importantly, SCANDARE is designed as a discovery-oriented biobanking infrastructure rather than a hypothesis-driven interventional trial. Biological samples are therefore not analyzed systematically at inclusion but are processed and analyzed through academic and industrial research collaborations, according to specific scientific questions, approved projects, and available technologies, in full compliance with patients’ consent.

All raw molecular data generated from SCANDARE samples are securely stored within the KDI storage system at Institut Curie, which supports standardized workflows, integration of multi-modal datasets, quality control, and long-term data integrity. Clinical data are collected from patients’ medical records and entered in a dedicated electronic Case Report Form (eCRF) designed for SCANDARE, ensuring structured and secure management of clinical variables. Access to SCANDARE samples and associated data is governed through a controlled authorization process involving the SCANDARE Principal Investigator and the research teams generating the data. Project-specific identifiers are used to anonymize datasets provided to collaborators, and published datasets are additionally deposited in controlled-access repositories, such as the European Genome-phenome Archive (EGA), in accordance with FAIR principles and patient consent.

### Ethical considerations and regulatory approvals

SCANDARE was approved by a national research ethics committee (CPP Ile-de-France 3) and the French National Agency for the Safety of Medicines and Health Products (ANSM) on December 09th, 2016. By participating in SCANDARE, patients received standard treatments and agreed to undergo additional sampling done along disease evolution.

Several amendments to the protocol have been made over the study and were approved by Ethics Committee (EC) and Agence nationale de sécurité du médicament et des produits de santé (ANSM). These amendments addressed regulatory and operational needs, including investigator updates, expansion of biospecimen collection, cohort extensions, inclusion of additional clinical sites, and adaptation of patient information and consent forms in compliance with the European General Data Protection Regulation (GDPR). A chronological overview of all major regulatory and ethics-related amendments, along with their operational implications, is provided in Table 1.

**Table 1:** Major regulatory and ethical amendments during the SCANDARE study

Date	Regulatory Authority	Amendment/Challenge	Operational impact
Sept 2017	EC, ANSM	Addition of new investigators	Expansion of recruiting clinical teams
July 2018	EC, ANSM	Introduction of blood and healthy tissue sampling; GDPR-compliant consent update	Extended biospecimen collection; re-consenting of participants
Sept 2020	EC, ANSM	Extension of HNSCC cohort (+ 100 patients)	Increased recruitment duration
Oct 2021	EC, ANSM	Extension of ovarian cancer cohort (+ 100 patients)	Increased recruitment duration
Feb 2023	EC, ANSM	Protocol update; addition of 3 external sites; creation of cervical cancer cohort	Multicenter expansion; new cancer indication
March 2022/March 2024	EC, ANSM	Update of declared investigators	Administrative compliance

To ensure ethical enrollment and minimize screening failures, patient inclusion was proposed by a physician before any intervention and only after histological confirmation. This physician-led policy reflects institutional and ethical guidelines, ensuring that patients are fully informed about the non-therapeutic nature of SCANDARE and that enrollment occurs in a manner consistent with their interests and consent.

All methods and study procedures were conducted in accordance with the Declaration of Helsinki, applicable French regulations governing biomedical research involving human participants, and the GDPR.

## Results

### Patient screening, enrollment, and follow-up

The implementation of an interventional, non-therapeutic study raised several issues (Table 1). Since patients do not expect to directly benefit from their participation in the study, as all interventions are non-therapeutic and samples are collected solely for research purposes, obtaining informed consent required careful explanation of the study objectives, potential risks, and the future use of their biological material and data [21]. The active involvement of the medical staff was critical in overcoming these challenges, as it enabled the timely identification of eligible patients during tumor boards, ensured that patients were fully informed about the non-therapeutic nature of the study, and facilitated obtaining consent prior to any surgical interventions. This workflow has facilitated inclusions over the years (Table 2).

**Table 2:** Challenges and solutions in terms of patient screening, enrollment, and follow-up

Topic	Context/Challenges	Solutions
Patient identification	• Screening to be performed before any therapeutic intervention in order to collect all needed samples	• Eligible patients should be identified during tumor boards by surgeons and physicians
Patient enrollment	• Study has to be proposed to patients by a physician before any intervention and a consent signed is mandatory in order to enroll patients	• Since patients might be at home when identified, the use of a teleconsultation and an electronic consent would facilitate the patient enrollment process
	• Diagnosis requires histological confirmation (e.g. patients with an ovarian mass on imaging) that is not immediately available	• The protocol should be flexible allowing inclusion after confirmation of diagnosis (during treatment, follow-up or recurrence), although not all samples would be available for future research
Patient follow-up	• The operational team (surgeons or radiologists, pathologists, clinical research associate, project manager) needs to be informed in case of recurrence in order to adequately collect samples	• A weekly manual check in the patient charts should be performed in order to not to miss any recurrence by the operational team

**Fig. 1:**
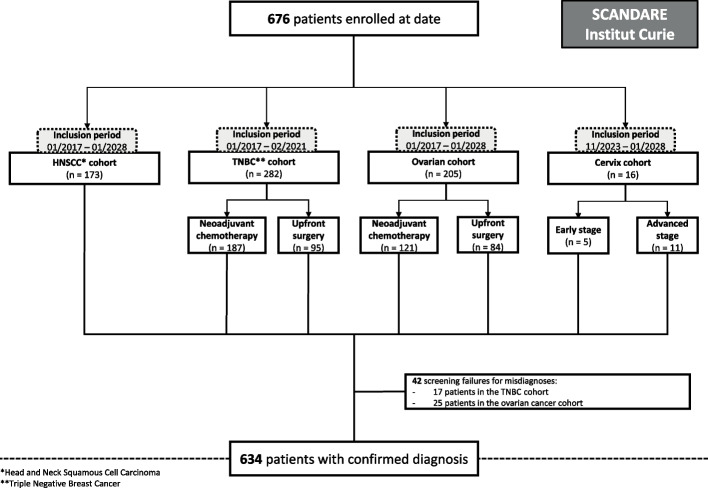
Consort diagram of the SCANDARE biobanking study

To date, 676 adult patients with newly diagnosed cancers were enrolled, including 173 patients with surgically resected head and neck squamous cell carcinoma (HNSCC), 282 patients with triple negative breast cancer (TNBC) (187 in a neoadjuvant chemotherapy [NAC] sub-cohort, and 95 patients treated with upfront surgery); 205 patients with ovarian cancer (121 in a neoadjuvant chemotherapy sub-cohort, and 84 patients treated with upfront surgery), and 16 patients with cervical cancer (5 early stage and 11 advanced stage). All participants provided signed informed consent. Among them, 42 patients withdrew from the study due to screening failure (17 in the TNBC cohort, and 25 in the ovarian cohort). In total, 634 patients were enrolled in SCANDARE with a confirmed diagnosis meeting the selection criteria (Fig. 1).

Key to our approach is the interventional study design, where patients provide explicit consent for their participation. Adapting to evolving regulatory requirements, regarding data protection, represented a major challenge in the development of SCANDARE. Multiple protocol amendments were required over time to ensure compliance with ethical and regulatory frameworks, including the implementation of the European General Data Protection Regulation (GDPR), expansion of biological sampling, cohort extensions, and inclusion of new clinical sites. These regulatory adaptations necessitated updates to patient information and consent forms, as well as re-consenting of previously enrolled participants, resulting in significant logistical efforts for the clinical research assistants. Information and consent forms were adapted to ensure patients’ active implication and approval in the future use of their samples and data beyond direct study objectives. A detailed overview of these regulatory milestones and amendments is provided in Table 1.

When it comes to patient enrollment, there are two primary obstacles. First, the study must be proposed by a physician before any intervention, with mandatory signed consent, and second diagnosis requires histological confirmation, which may not be immediately available. To overcome these issues, teleconsultation and electronic consent might facilitate the enrollment process, especially since patients are usually at home when identified. In such cases, the physician explains the study protocol remotely, and the patient brings the signed paper consent on the day before surgery during hospital admission. Additionally, the protocol should allow for patient inclusion after diagnosis confirmation, whether during treatment, follow-up, or recurrence, though not all samples may be available for future research (Table 2).

For patient follow-up, the challenge is ensuring that the operational team (including surgeons, radiologists, pathologists, clinical research associates and the operational team) is informed of any recurrence to collect samples appropriately. This can be managed by implementing a weekly manual check of patient charts to ensure that no recurrence is missed, although it requires significant staffing resources (Table 2). This procedure was actively implemented by the SCANDARE study team throughout the study period.

In a multicenter setting, coordinating patient identification, consent, longitudinal follow-up, and sample collection across sites added further operational complexity. These challenges were addressed through close coordination between clinical teams, clinical research associates, and centralized operational staff, ensuring consistent application of study procedures and timely sample collection across all participating centers. In addition, biological samples collected at external sites are periodically transferred to Institut Curie after every 30–50 inclusions, enabling centralized storage, quality control, and more efficient access to samples for collaborations.

Integration of biobank sampling into the routine patient journey minimized direct interventions and contributed to patient acceptance. Initially, we expected some physicians’ hesitations about the SCANDARE project precisely due to its non-therapeutic nature. However, patient acceptance combined with the quick emergence of research projects capitalizing on SCANDARE data all contributed to overcoming initial hesitations. These projects, spanning 35 academic and industrial collaborations [20, 22–30], addressed key translational research themes and helped demonstrate the scientific value of SCANDARE, reinforcing physician and institutional support.

### Samples collection, processing, and storage

SCANDARE workflow implementation required considerable perseverance. Once these challenges were addressed, a lot of interest emerged, driven by the various opportunities offered by this innovative prospective longitudinal interventional design.

This study longitudinally collects and preserves high-quality tumor and blood samples guaranteed by a collection, processing and storage on-site, in real time by certified platforms that apply standard operating procedures (Table 3).

**Table 3:** Challenges and solutions in terms of samples collection, processing, and storage

Topic		Context/Challenges	Perspectives/Solutions
All samples (blood & tissue)		• High-quality samples, processing and storage need to be collected at different time points • Sample collection should be performed during routine patients visits	• Samples must be collected, processed and stored on-site in real time • Samples processing and storage should be performed by certified platforms that apply standard operating procedures, including traceability • Sample collection time points should be decided based on routine hospital visits
Blood Samples		• Different types of blood products can be biobanked following real time processing, including plasma, serum, buffy coat, PBMC and whole blood	• Blood must be collected in dedicated sampling tubes (EDTA for whole blood and buffy coat, dry tube for serum, Streck tube for ctDNA, and CPT™ tube for PBMC) • Blood must be adequately processed on real time
Tissue samples	All (FFPE, frozen, and fresh)	• Samples collected during surgery or interventional radiology are primarily used for diagnosis • The collection of research samples as part should not jeopardize routine care • The time between sample collection and biobanking sometimes needs to be short to preserve cell viability and guarantee high quality analyses • Depending on the sample size collected, it may not always be possible to achieve all preservation types needed	• All tissue samples should first be characterized by a pathologist in order to confirm diagnosis, cellularity and the type of samples to be selected for biobanking (tumoral/juxta-tumoral/metastasis) • If the expected sample size is too small (e.g. laparoscopy, ultrasound microbiopsy), several fragments from a single sample can be collected to enable all preservation types needed • Samples should be stored in adequate medium until biobanking to preserve cell viability • Depending on the size of the research samples collected, a prioritization plan should be available in order to know what type of preservation should be chosen
	Fresh samples	• Fresh samples have to be processed in real time	• Research teams have to be ready to receive samples collected at the scheduled surgery/biopsy time to enable analyses in real time for translational research • Workflow set up must be fluid between the various partners involved (research and care teams) to ensure fast and high-quality samples collection and processing

**Fig. 2:**
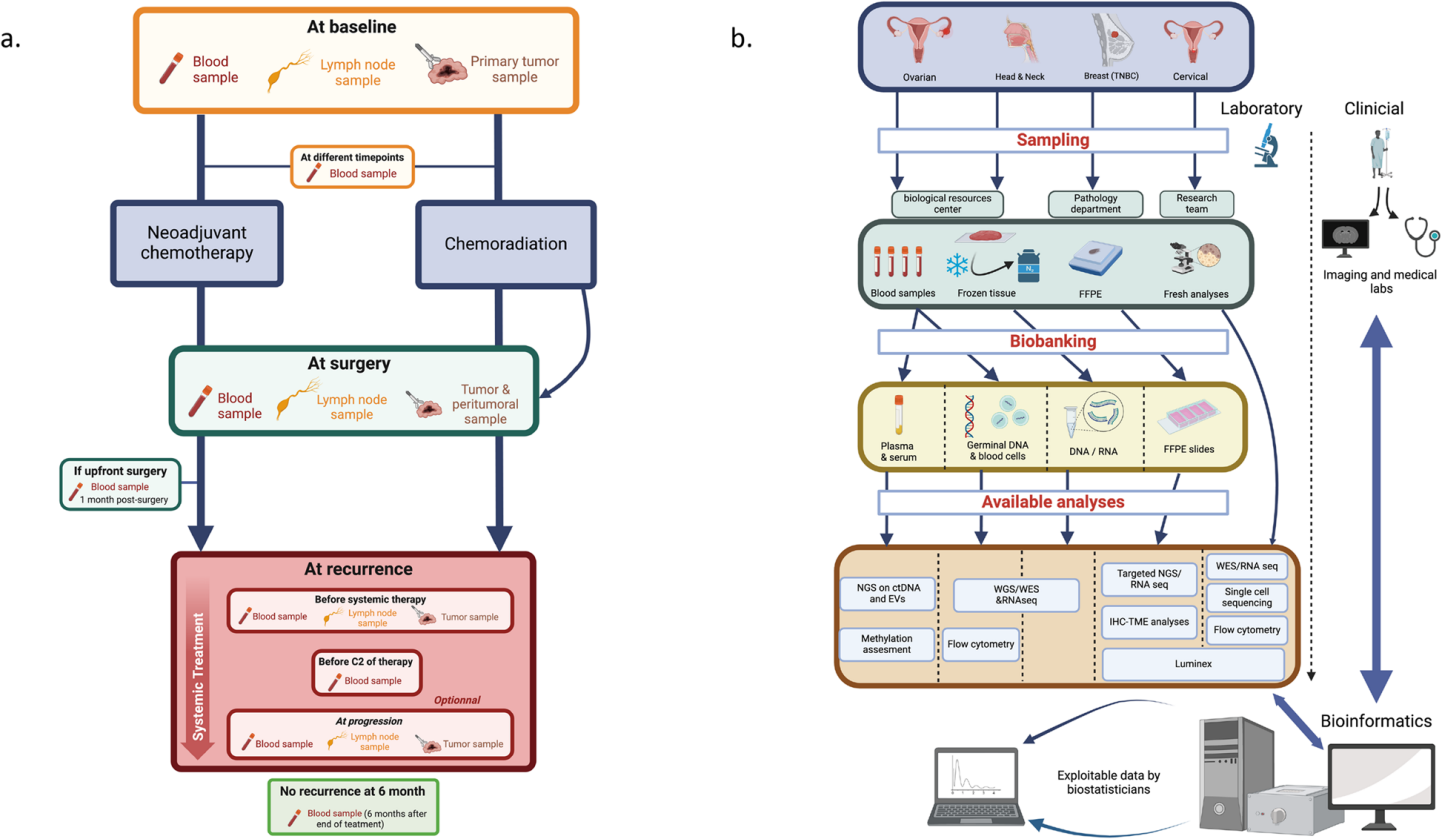
SCANDARE (**A**) sampling design, and (**B**) samples and data workflow

Tissue and blood samples are longitudinally collected at two sites of Institut Curie (Paris and Saint-Cloud) for ovarian, TNBC and HNSCC cohorts, and at 3 sites (Institut Curie Paris, Institut Curie Saint-Cloud and Oscar Lambret center) for the cervical cancer cohort. Tissue samples (tumoral, juxta-tumoral, and lymph nodes; fragments: Min: 1 – Max: 14 per sample biobanked) are collected in frozen, Formalin-Fixed Paraffin-Embedded (FFPE), and fresh forms at baseline, post-neoadjuvant chemotherapy surgery (post-NAC), at recurrence, and at disease progression. Depending on the cohorts, blood (including whole blood, plasma, serum, PBMC, and buffy coat; aliquots of 2 mL: Min: 1 – Max: 18 mL per sample biobanked) is collected at baseline, during NAC treatment (before the 2nd cycle), post-NAC, post-surgery, after chemoradiotherapy (2 months post chemoradiotherapy), 6 months post-surgery/chemoradiotherapy, at recurrence (first loco-regional or distant recurrence or time of failure to complete curative-intent treatment), during treatment initiated for a recurrence (before the 2nd cycle of treatment), and at disease progression (progressive disease following the treatment initiated for a recurrence) (Fig. 2A).

Between January 2017 and May 2024, blood samples were collected from 170 HNSCC patients (Supplementary Fig. 1 A), 225 TNBC patients (88 upfront surgery, and 167 NAC) (Supplementary Fig. 2 A), 179 ovarian cancer patients (68 upfront surgery, and 111 NAC) (Supplementary Fig. 3 A), whereas tissue samples were collected from 163 HNSCC patients (Supplementary Fig. 1B), 240 TNBC patients (71 upfront surgery and 169 NAC) (Supplementary Fig. 2B), and 177 ovarian cancer patients (68 upfront surgery, and 109 NAC) (Supplementary Fig. 3B). 

The cervical cancer cohort opened more recently. Between November 2023 and May 2024, blood and tissue samples were collected from 15 patients across the 3 different sites (Supplementary Fig. 4).

Quantitative indicators of workflow performance demonstrate high initial success rates for sample collection at baseline. Specifically, 79–98% of scheduled blood samples were successfully collected across all cohorts and sample types (PBMC, plasma, whole blood, serum), while tissue samples achieved 80–95% success in frozen and FFPE preservation. Fresh tissue samples showed slightly lower success rates (65–80%) due to limitations in sample availability and quality.

During longitudinal follow-up, biobanking success rates decreased progressively, with overall rates ranging from 10 to 50% depending on timepoint, cohort, and sample preservation type. These figures reflect the inherent challenges of maintaining high-quality longitudinal sampling in a multicenter, prospective biobanking study (Supplementary Fig. 1–4). Despite these difficulties, the SCANDARE workflow enabled consistent processing, storage, and integration of biological samples, ensuring robust datasets for translational research.

Several challenges emerged while setting up the SCANDARE study. Bridging clinical and research teams in real-time necessitated optimized sample and data workflows that can only be done through a fluid communication between researchers and clinicians (Table 3). To achieve this, SCANDARE sampling was carried out at specific timepoints during the patient's disease journey, such as at diagnosis, during surgery, and at recurrence if any. All tissue specimens are first reviewed by a pathologist for routine diagnostic purposes, after which selected fragments are allocated to SCANDARE for biobanking. This expert-driven selection ensures the presence and relevance of tumor material and thereby guarantees the quality and suitability of the samples for future translational and molecular analyses. In cases where no residual tumor cells are identified, only juxta-tumoral tissue is preserved. Consequently, storing a sample may not always be feasible depending on the size of the collected tissue. In the context of biopsies, several fragments from the same sample can be independently collected for diagnosis on one hand and for biobanking study on the other (Table 3). Moreover, this alignment with traditional diagnostic routines significantly enhances efficiency, saves resource utilization, and thereby bolsters the sustainability of the biobanking process. Yet, while collecting samples at inclusion presented no logistical hurdles, maintaining meticulous adherence to schedule for subsequent sampling required strict coordination, especially at recurrence (Table 3). Failure to do so may lead to a marked decrease in the number of successfully completed sampling at recurrence or later, a challenge faced in a different longitudinal study [13].

SCANDARE workflow includes real time analyses on fresh tissues and blood samples while other samples are processed for preservation and future analyses. A dual preservation strategy is employed, encompassing formalin and paraffin fixation (FFPE) techniques for maintaining tissue morphology, and immediate freezing to ensure long-term molecular stability. Capitalizing on the infrastructure of the pathology department and the biological resources center at Institut Curie, processing and storage can be carried out on-site under optimal quality conditions, helping to produce solid scientific results (Table 3). To ensure harmonized implementation across all participating sites, including external centers, a standardized laboratory manual detailing cohort- and timepoint-specific sampling procedures and storage protocols was developed and disseminated throughout the SCANDARE network. This harmonization was critical to mitigate site-to-site variability, ensure comparable sample quality, and maintain the integrity of downstream translational analyses in a multicenter context.

Analyses on fresh samples provides real-life insights of tumor microenvironment interactions and is one of the main objectives of SCANDARE, including single-cell RNA sequencing, development of *in vivo* models (patient-derived xenograft, PDX), *ex vivo* models (organoids, patient-derived tumor fragments, PDTFs), and immunophenotyping using flow cytometry (FACS). Simultaneously, preserving samples in FFPE format and freezing them ensures the reliability and consistency of future analyses and ensures the longevity of SCANDARE. Performing a wide range of analyses, from histopathology to genomics and proteomics, and managing the meticulous conservation processes require streamlined workflows, well-equipped wet labs, and sophisticated technical platforms. Nevertheless, it should be emphasized that while setting up such an efficient infrastructure certainly requires initial investment, its subsequent operational efficiency makes it adaptable and applicable to various cancer types.

To address the challenge of real-time processing of fresh tissue and blood, it is possible to adapt the preservation methods. Fresh tissue can be stored overnight in medium at 4 °C to maintain cell viability, and blood can be collected in Streck or CPT™ tubes to preserve ctDNA and PBMCs respectively, providing researchers with greater flexibility for conducting analyses (Table 3).

### Data generation and management

Clinical data are collected from patients' medical records and are filled in an electronic Case Report Form (eCRF) dedicated to SCANDARE. The list of clinical variables of interest evolves with innovations and their availability is needed for translational research purposes. For this, a flexible eCRF must be implemented to allow the long-term integration of these new relevant clinical data (Table 4).

**Table 4:** Challenges and solutions in terms of data generation and management

Topic	Context/Challenges	Perspectives/Solutions
Clinical data	• A minimum dataset of clinical variables is often necessary for translational research • Novel clinical data not collected initially may eventually become necessary	• An eCRF dedicated to the biobanking study should be set up to facilitate the clinical data and samples annotations extraction • eCRF should be flexible in order to easily be able to add new variables
Data generation	• Data generation is linked to the objectives of the biobanking study yet techniques evolve • Different types of data (including omics and imaging) are generated across research projects	• The primary objective of the biobanking study should be broad enough to allow the generation and further utilization of omics data from different technologies • A dedicated platform should be developed in order to centralize and share data generated
Omics data	• Different omics data must be stored on a secured platform that needs to be accessed by different people	• A dedicated platform should be developed in order to allow to securely store a large volume of raw data and be accessible by authorized collaborators (both internally & externally)
FFPE slides	• Tissue slides might be stained for diagnosis and/or translational research analyses • Slides are sometimes numerized for data storage	• Numerized slides should be stored in a secure repository with access restricted to authorized collaborators (both internally & externally)
Data analyses	• Data generated from different omics have to be used for analyses that meet the objectives of the biobanking study	• Bioinformatics tools and the types of analyses to be carried out should be adapted to the objectives of the biobanking study

The design is tailored to enable the biobanking and further comprehensive characterization of any cancer type ensuring that genomic, transcriptomic, epigenomic, immune and other data can be made available when needed. All available pre-specified analyses are summarized in Fig. 2B. The remarkable flexibility of SCANDARE is key. Since patient consent is secured through the interventional design, numerous analyses are generated on the collected samples, making it a versatile research platform (Table 4).

Efficient and standardized processes for data storage and integration were facilitated using knowledge gained from previous experiences at the Institut Curie. A seamless information system named KDI that fully supports the essential bioinformatics requirements for precision oncology projects was previously used for SHIVA01 (NCT01771458) or Bio-RAIDs (NCT02428842) studies in order to facilitate omics data management [31, 32]. However, KDI collects exclusively omics data. Storage of images, including slide digitization, remains a challenge. To address this limitation, the development of a dedicated repository is recommended (Table 4), although this has not yet been implemented across the SCANDARE network. Translational studies of the PEVOsq European trial (NCT04357873) is an example, in which a similar approach was implemented to efficiently integrate multi-omics data, digitized slides and clinical data generated by various partners in an integrative dedicated platform [33].

All raw molecular data generated from SCANDARE samples are considered part of Institut Curie’s intellectual property and are securely stored within the KDI infrastructure. Data access is governed through a controlled authorization process involving the SCANDARE Principal Investigator and the research teams that generated the data. When datasets are published, corresponding data are additionally deposited in repositories such as the European Genome-phenome Archive (EGA) for which control access is managed by the Institut Curie Data Access Committee (DAC), in accordance with FAIR principles and patient consent.

SCANDARE's unique regulatory framework, with its forward-looking and longitudinal approach, is well-suited to the growing interest in translational research within academic and industry. This framework has supported 35 projects to date [20, 22–30] spanning diverse translational research themes, demonstrating its utility in fostering high-quality collaborations while respecting patient consent. Efficient and secure data sharing is essential for effective collaborations, ensuring valuable information is exchanged seamlessly while maintaining high standards of confidentiality. Data anonymization procedures are customized for each collaboration based on scientific objectives and contracts, ensuring patient consent is respected. For example, Patients IDs can be uploaded in secured storage bucket with restricted access to IT teams only. Consequently, a corresponding project-specific ID can be generated. This unique ID will be used throughout the entire project when data are provided to collaborators to perform analyses. This procedure facilitates the anonymization protocol at the end of the project.

Managing the generated data involves significant logistical efforts, including maintaining data integrity, ensuring cost-effective storage, and accommodating large data transfers. Interoperability issues arise from the varied data analysis software used, requiring standardization and formatting for effective sharing. Data protection is central to all processes involved in running the biobank, integrating all stakeholders [34].

Despite these complexities, we have established efficient and standardized processes, demonstrating the feasibility of an institutional biobanking study with rigorous standards for data privacy and management. These workflows have required a great deal of upstream work but can now be exploited for future projects (Table 4).

### Analyses performed for precision medicine across collaborations

**Fig. 3:**
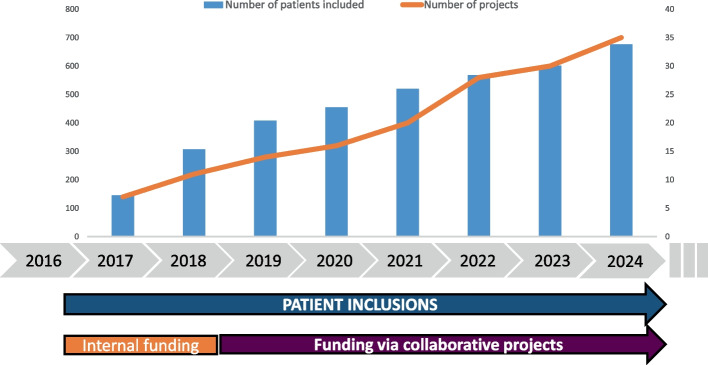
Number of patients included in SCANDARE and number of projects using SCANDARE samples over time

This dynamic biobanking study generated longitudinal tumor and blood samples and curated omics and clinical data, supporting an increase from 7 collaborations in 2017 to 35 collaborations in 2024 (27 academic, 8 industrial), illustrating its utility for translational research projects (Fig. 3). From the outset, samples and data generated from the biobank naturally attracted academic partnerships due to its institutional nature but also industrial partnerships and collaborations acknowledging the advantages of the design and its meticulously curated database.

SCANDARE provides diverse longitudinal samples and curated omics data across multiple cancer types. Moreover, SCANDARE's first patients’ data sets are now accessible for conducting statistical analyses (including survival outcome) and can be reused in compliance with patients’ consent within a strict and secure regulatory framework.

This growth trajectory is evidenced by 35 active academic and industrial collaborations to date, encompassing a variety of translational research projects in precision medicine and guaranteeing sustainable long term benefits securing the study costs.

These projects address key themes including circulating tumor DNA–based disease monitoring, intra-tumor heterogeneity and spatial profiling, transcriptomic and epigenomic biomarkers of prognosis and treatment response, tumor microenvironment characterization, and methodological developments for high-dimensional data analysis across cancer types.

Data generation and analyses performed across these collaborations have been presented at world-renowned international conferences such as the AACR (American Association for Cancer Research) and ESMO (European Society for Medical Oncology) and disseminated through numerous publications, both published and in progress [20, 22–30].

## Discussion

SCANDARE is a dynamic biobanking study, initiated by Institut Curie, Paris, France aimed at bridging the gap between basic research and clinical applications in the field of precision oncology. The rapid evolution of Next Generation Sequencing (NGS) and novel technologies has revolutionized cancer treatment paradigms, enabling the development of targeted therapies and immunotherapies tailored to specific molecular alterations [1–3]. This study underscores the importance of translational research in leveraging these technological advances to improve clinical outcomes in HNSCC, TNBC, ovarian and cervical cancers patients.

A key strength of SCANDARE lies in its dynamic and prospective design, which allows for the longitudinal collection of biological samples and clinical data from patients with newly diagnosed cancers. By focusing on tumor molecular alterations, the tumor microenvironment, and immune parameters, SCANDARE aims to unravel complex interactions that influence oncogenesis, treatment response and resistance mechanisms, in link with the emergence of tissue-agnostic therapies [4, 5].

The implementation of SCANDARE has been challenging, particularly in regarding regulatory compliance, patient consent and the organization of standardized workflows for sample collection, processing, storage, and data management. These challenges were actively addressed by the study team through measures such as the implementation of GDPR, multiple protocol amendments with re-consenting of patients, pathologist-led selection of tissue fragments for biobanking, weekly manual review of patient charts to track recurrences, the dissemination of standardized laboratory manuals across all participating sites, and the use of the KDI information system to manage omics data efficiently. These operational strategies ensured regulatory adherence, high-quality sample collection, and harmonized workflows across the network. The successful implementation of these procedures is further illustrated by the 35 academic and industrial research projects [20, 22–30] that have capitalized on SCANDARE data, demonstrating the practical utility of the framework for translational research.

SCANDARE demonstrates that longitudinal, prospective biobanking studies can effectively overcome these regulatory and operational challenges while generating high-quality samples suitable for a wide range of translational research applications. Expert-driven tissue selection, integration of sampling into routine clinical care, and several preservation strategies (fresh, fixed and frozen) guaranteed sample integrity for genomics, transcriptomics, proteomics, single cell and immune profiling. The impact of these strategies is illustrated by the 35 academic and industrial projects that have used SCANDARE data [20, 22–30], highlighting the practical utility of the framework and its contribution to advancing precision oncology.

The successful integration of SCANDARE within routine clinical workflows has not only ensured high-quality longitudinal sample collection and robust regulatory compliance, but also demonstrated a tangible return on investment, as the infrastructure and procedures established have enabled multiple collaborations, generating valuable translational data while maintaining financial and operational sustainability. These collaborations prove to be an exceptional opportunity as they transform the SCANDARE project into a robust business model, capable of self-sustaining and making the initial investment fruitful. By remaining as close as possible to the routine oncology patient journey, logistical constraints were reduced and real world data collected at key timepoints in the course of the disease are now available.

Building on this success, the study is designed to evolve further. Future developments include the expansion to additional national and European centers, allowing increased patient inclusion and broader representation of cancer subtypes, like other relevant initiatives such as "All of Us" and "Cancer Moonshot" [11, 12]. Beyond tumor tissue and blood, the integration of additional biospecimen such as saliva, feces, and urine can be collected to enable more comprehensive analyses. Increased longitudinal tumor sampling, particularly at relapse and progression, will further strengthen investigations into tumor evolution and resistance mechanisms.

Moreover, SCANDARE provides comprehensive and well-curated data generated from a variety of complementary technologies, which, together with its broad objectives, has attracted numerous academic and industrial collaborators who have generated or accessed these datasets. This distinguishes SCANDARE from other clinical biobank studies, which generally focus on answering very specific scientific questions [18, 35]. As the project evolved, it gained strength and self-sufficiency, allowing for the exploration of samples and data with patients' consent to address infinite scientific and medical inquiries.

Building on this foundation, SCANDARE aims to substantially expand the generation of multi-modal molecular data, integrating genomics, transcriptomics, proteomics, spatial profiling, and immune phenotyping. The harmonized and well-annotated nature of these datasets provides a unique opportunity for large-scale data integration and for the development of artificial intelligence and machine-learning models to identify predictive biomarkers, stratify patients, and support clinical decision-making. By facilitating access to these high-quality datasets for the scientific community, SCANDARE promotes collaborative research and maximizes the translational value of collected samples.

In conclusion, SCANDARE is a dynamic and longitudinal biobanking study that has enabled the data generation which represents a robust database reflecting real-world evidence. Its continued expansion in scope, geography, and analytical depth positions SCANDARE as a valuable infrastructure for pan-cancer translational research at both national and European levels.

## Supplementary Information

Supplementary Material 1.

Supplementary Material 2.

Supplementary Material 3.

Supplementary Material 4.

Supplementary Material 5.

## Data Availability

Original files and raw data files from the SCANDARE study will be made available from the corresponding authors upon reasonable request. The data are not publicly available due to information that could compromise the privacy of the research participants.
