# Supplementary material for: SCANDARE: an institutional dynamic prospective interventional biobanking study
Source: BMC Cancer. 2026 Feb 5;26:330. doi: 10.1186/s12885-026-15680-5 (PMC12977387; doi:10.1186/s12885-026-15680-5)
Supplement: Supplementary file 1 — Supplementary Material 1. [file 12885_2026_15680_MOESM1_ESM.pdf]

A

Upfront surgery (n= 170)

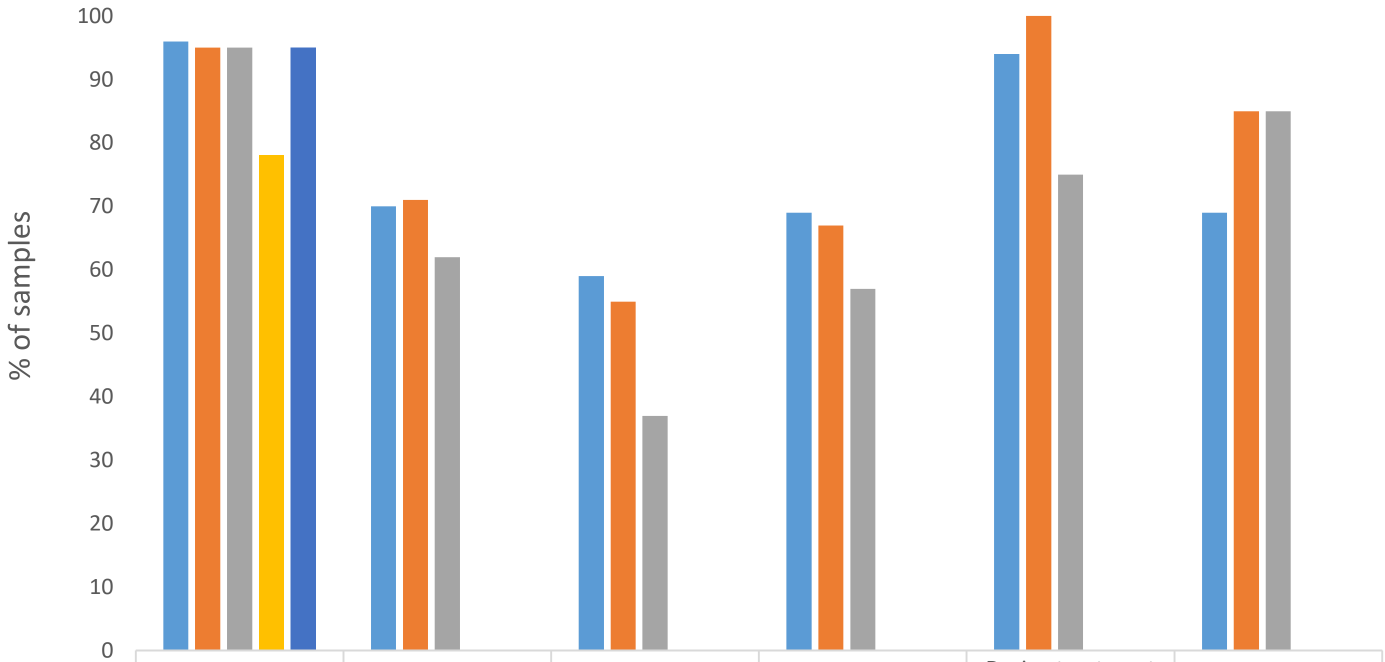

	At baseline	Post-surgery	6 months post-surgery	At recurrence	During treatment following recurrence	At progression
Plasma	96	70	59	69	94	69
Serum	95	71	55	67	100	85
PBMC	95	62	37	57	75	85
Buffy coat	78					
Whole blood	95					

B

Upfront surgery (n= 163)

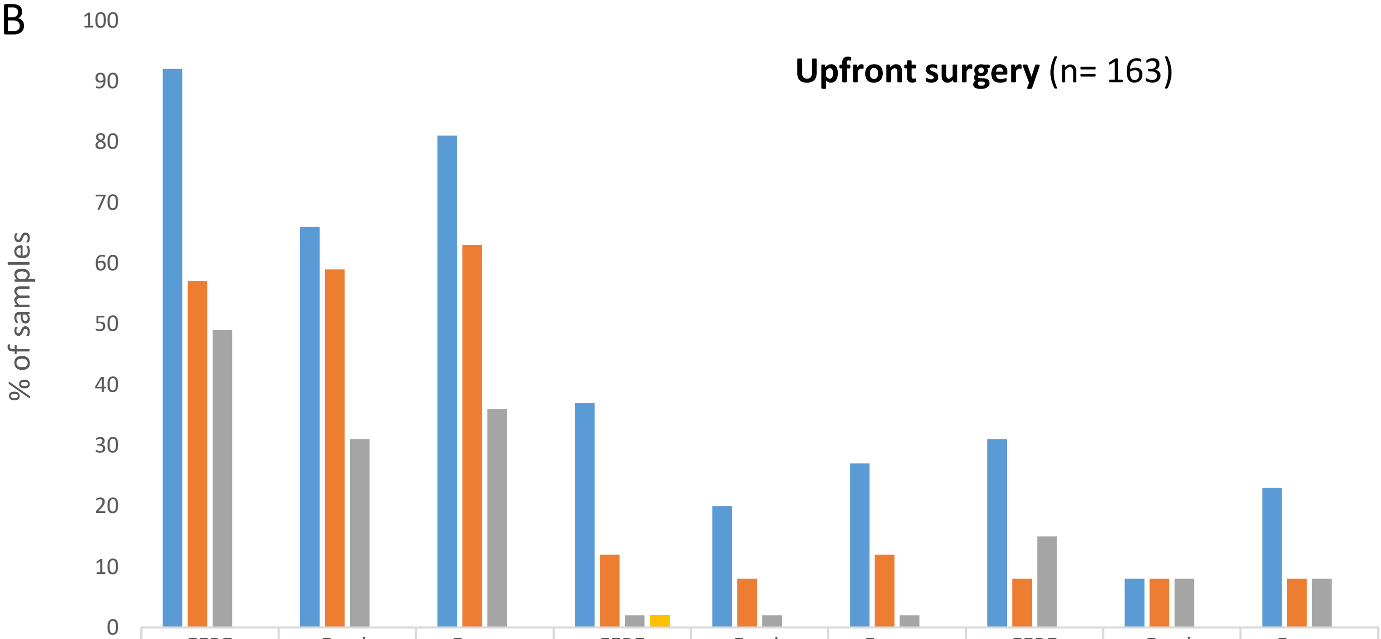

	At baseline			At recurrence			At progression		
	FFPE	Fresh	Frozen	FFPE	Fresh	Frozen	FFPE	Fresh	Frozen
Tumor	92	66	81	37	20	27	31	8	23
Juxta-tumoral	57	59	63	12	8	12	8	8	8
Lymph node	49	31	36	2	2	2	15	8	8
Metastase				2					

Supplementary Figure 1 : Proportion of patients with available blood (A) and tissue (B) samples obtained in the HNSCC cohort
