# Supplementary material for: SCANDARE: an institutional dynamic prospective interventional biobanking study
Source: BMC Cancer. 2026 Feb 5;26:330. doi: 10.1186/s12885-026-15680-5 (PMC12977387; doi:10.1186/s12885-026-15680-5)
Supplement: Supplementary file 2 — Supplementary Material 2. [file 12885_2026_15680_MOESM2_ESM.pdf]

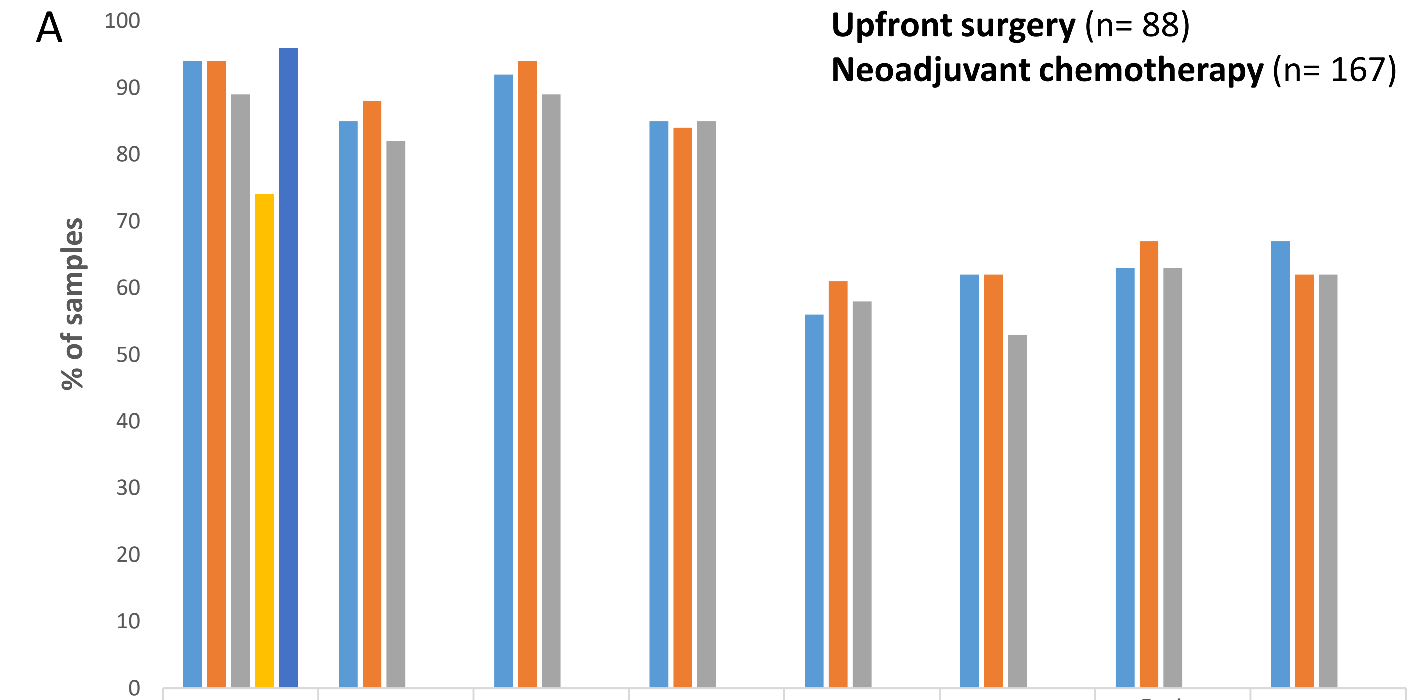

	At baseline	Post-surgery	During NAC	Post-NAC	6 months post-surgery	At recurrence	During treatment following recurrence	At progression
Plasma	94	85	92	85	56	62	63	67
Serum	94	88	94	84	61	62	67	62
PBMC	89	82	89	85	58	53	63	62
Buffy coat	74							
Whole blood	96							

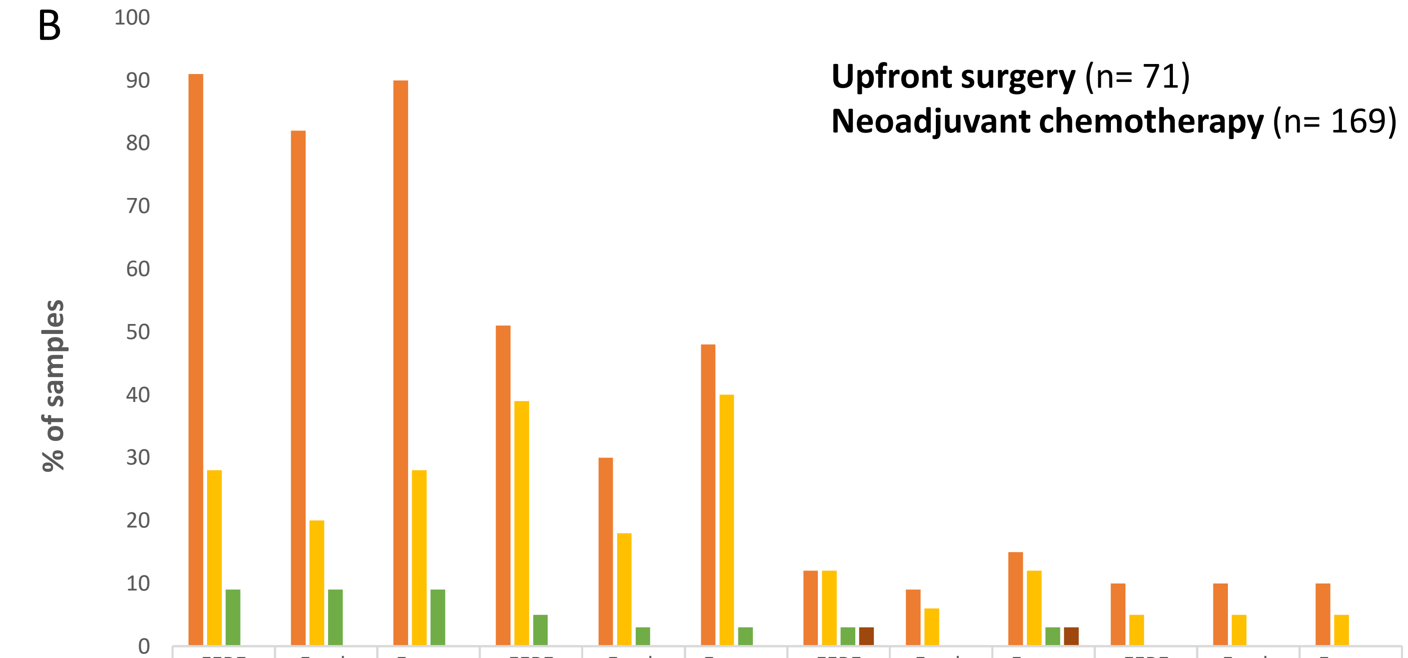

	At baseline			Post-NAC			At recurrence			At progression		
	FFPE	Fresh	Frozen	FFPE	Fresh	Frozen	FFPE	Fresh	Frozen	FFPE	Fresh	Frozen
Tumor	91	82	90	51	30	48	12	9	15	10	10	10
Juxta-tumoral	28	20	28	39	18	40	12	6	12	5	5	5
Lymph node	9	9	9	5	3	3	3		3			
Metastase							3		3			

Supplementary Figure 2 : Proportion of patients with available blood (A) and tissue (B) samples obtained in the TNBC cancer cohort
