# Supplementary material for: SCANDARE: an institutional dynamic prospective interventional biobanking study
Source: BMC Cancer. 2026 Feb 5;26:330. doi: 10.1186/s12885-026-15680-5 (PMC12977387; doi:10.1186/s12885-026-15680-5)
Supplement: Supplementary file 3 — Supplementary Material 3. [file 12885_2026_15680_MOESM3_ESM.pdf]

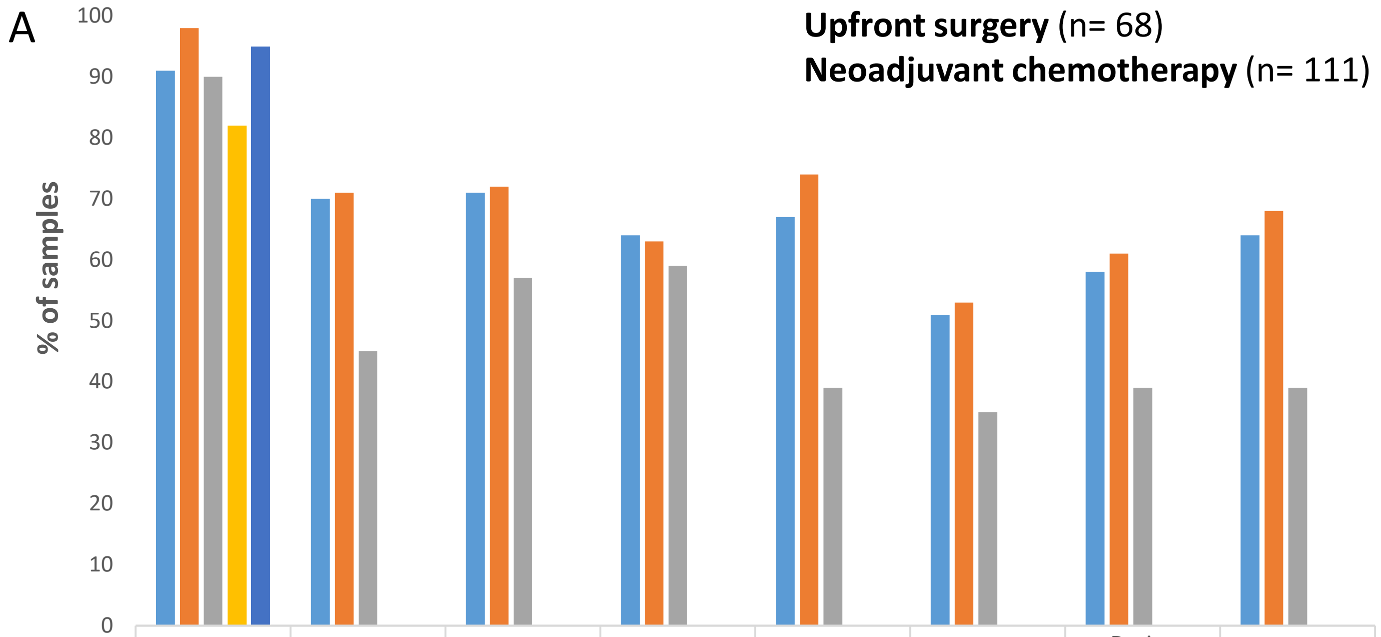

	At baseline	Post-surgery	During NAC	Post-NAC	6 months post-surgery	At recurrence	During treatment following recurrence	At progression
Plasma	91	70	71	64	67	51	58	64
Serum	98	71	72	63	74	53	61	68
PBMC	90	45	57	59	39	35	39	39
Buffy coat	82							
Whole blood	95							

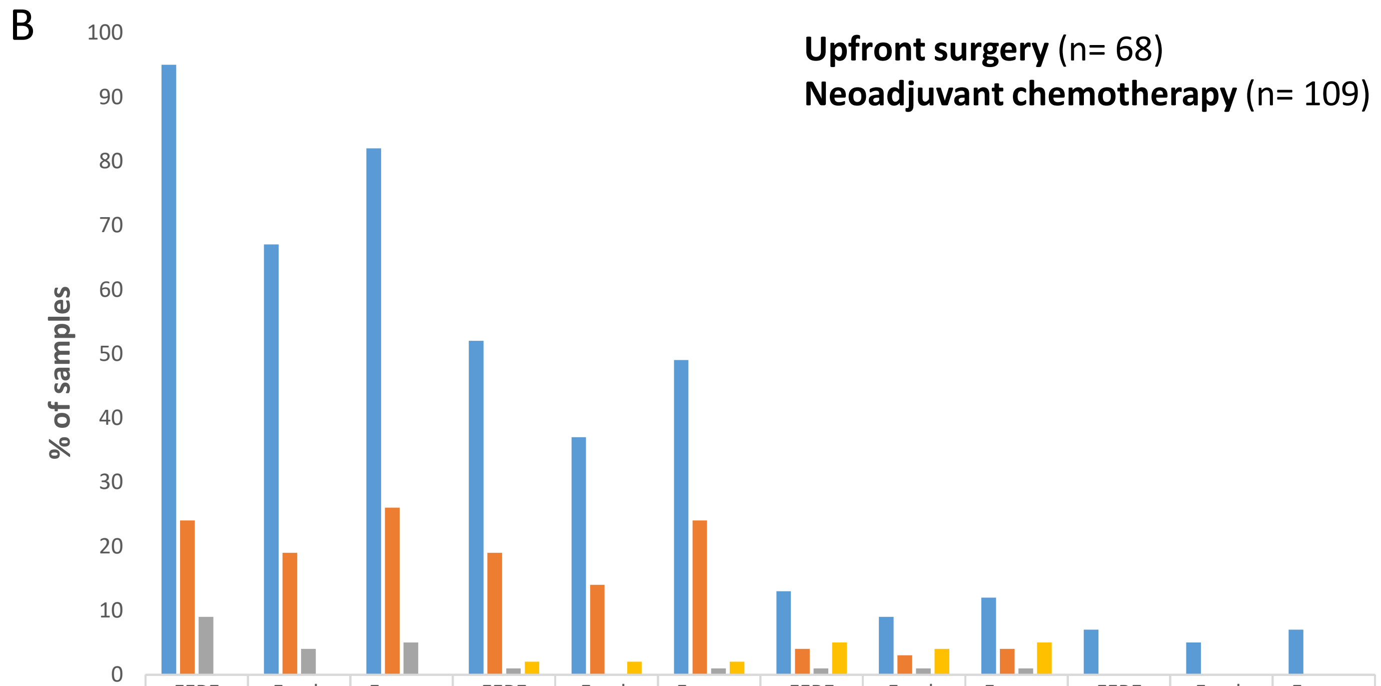

	At baseline			Post-NAC			At recurrence			At progression		
	FFPE	Fresh	Frozen	FFPE	Fresh	Frozen	FFPE	Fresh	Frozen	FFPE	Fresh	Frozen
Tumor	95	67	82	52	37	49	13	9	12	7	5	7
Juxta-tumoral	24	19	26	19	14	24	4	3	4			
Lymph node	9	4	5	1		1	1	1	1			
Metastasis				2	2	2	5	4	5			

Supplementary Figure 3 : Proportion of patients with available blood (A) and tissue (B) samples obtained in the ovarian cancer cohort
