# Supplementary material for: SCANDARE: an institutional dynamic prospective interventional biobanking study
Source: BMC Cancer. 2026 Feb 5;26:330. doi: 10.1186/s12885-026-15680-5 (PMC12977387; doi:10.1186/s12885-026-15680-5)
Supplement: Supplementary file 4 — Supplementary Material 4. [file 12885_2026_15680_MOESM4_ESM.pdf]

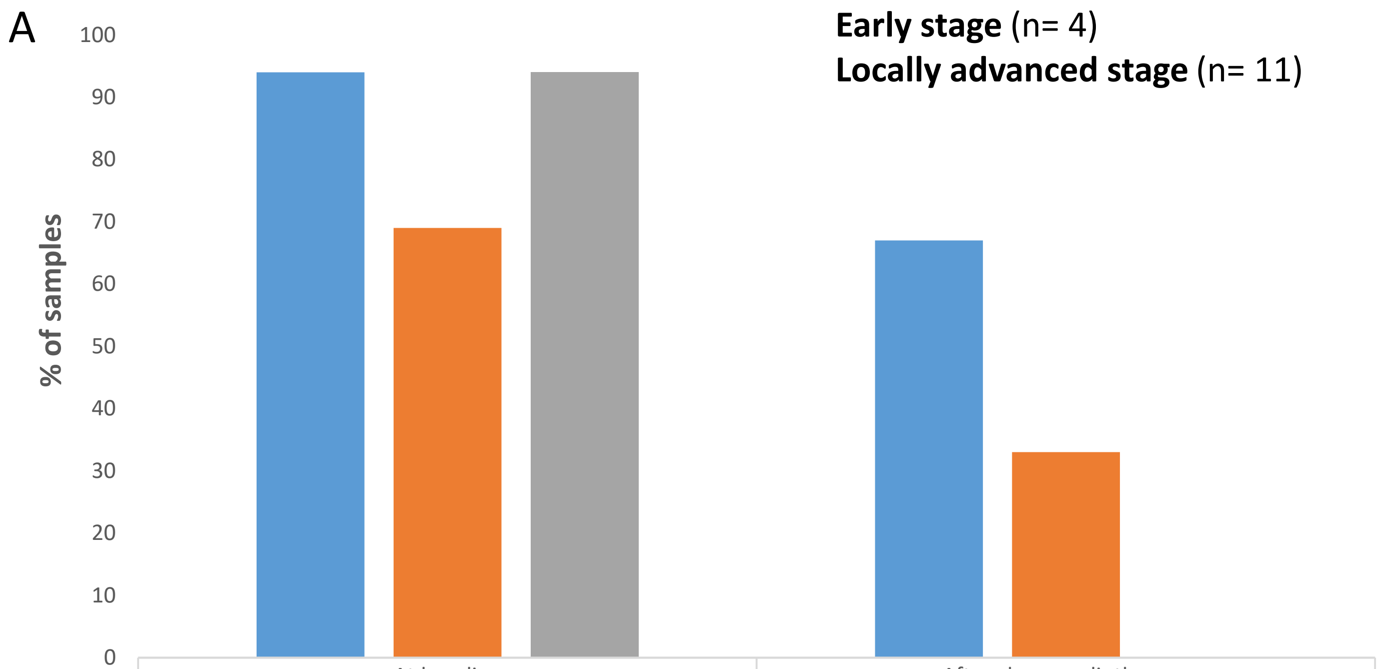


| | At baseline | After chemoradiotherapy |
|---|---|---|
| Plasma | 94 | 67 |
| Serum | 69 | 33 |
| Whole blood | 94 | |

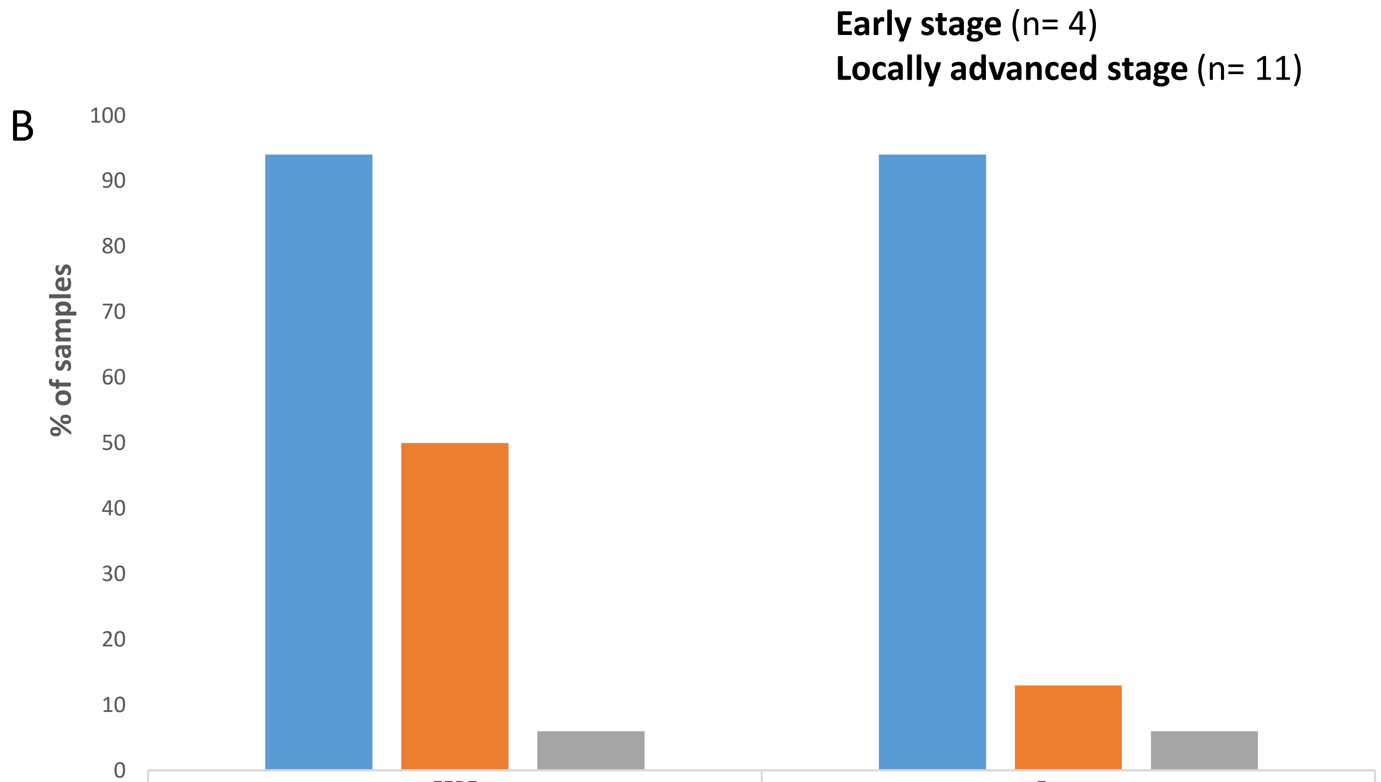


| | FFPE | Frozen |
|---|---|---|
| | At baseline | |
| Tumor | 94 | 94 |
| Lymph node | 50 | 13 |
| Juxta-tumoral | 6 | 6 |

*Supplementary Figure 4 : Proportion of patients with available blood (A) and tissue (B) samples obtained in the cervical cancer cohort*
