# Supplementary material for: SCANDARE: an institutional dynamic prospective interventional biobanking study
Source: BMC Cancer. 2026 Feb 5;26:330. doi: 10.1186/s12885-026-15680-5 (PMC12977387; doi:10.1186/s12885-026-15680-5)
Supplement: Supplementary file 5 — Supplementary Material 5. [file 12885_2026_15680_MOESM5_ESM.docx]

**Supplementary Methods**

Supplementary Figures 1, 2, 3 and 4 show an overview of blood and tissue samples biobanked in SCANDARE from January 2017 to May 2024. The percentage of samples biobanked has been calculated for each time point as described below:

- **At baseline**: It corresponds to the total number of patients with a sample collected at baseline out of the total number of patients enrolled in the study with a confirmed diagnosis.
- **Post-surgery**: It corresponds to the total number of patients with a sample collected post-surgery out of the total number of patients with a confirmed diagnosis and still included in the study at this time point.
- **During neoadjuvant chemotherapy (NAC)**: It corresponds to the total number of patients with a sample collected during NAC out of the total number of patients with a confirmed diagnosis who received NAC and still included in the study at this time point.
- **Post-NAC**: It corresponds to the total number of patients with a sample collected at post-NAC out of the total number of patients with a confirmed diagnosis who had a post-NAC surgery and still included in the study at this time point.
- **After chemoradiotherapy**: It corresponds to the total number of patients with a sample collected 2 months after chemoradiotherapy out of the total number of patients with a confirmed diagnosis who received chemoradiotherapy and still included in the study at this time point.
- **6 months post-surgery/chemoradiotherapy**: It corresponds to the total number of patients with a sample collected at 6 months post-surgery/chemoradiotherapy out of the total number of patients with a confirmed diagnosis, a follow-up of 6 months from surgery/chemoradiotherapy and still included in the study at this time point.
- **At recurrence**: It corresponds to the total number of patients with a sample collected at recurrence out of the total number of patients with a confirmed diagnosis, who experienced a recurrence and still included in the study at this time point.
- **During treatment following recurrence**: It corresponds to the total number of patients with a sample collected during treatment following recurrence out of the total number of patients with a confirmed diagnosis, who received treatment following recurrence and still included in the study at this time point.
- **At progression**: It corresponds to the total number of patients with a sample collected at progression out of the total number of patients with a confirmed diagnosis, who experienced a progression and still included in the study at this time point.
